# Image Registration in Longitudinal Bone Assessment Using Computed Tomography

**DOI:** 10.1007/s11914-023-00795-6

**Published:** 2023-06-02

**Authors:** Han Liu, Pholpat Durongbhan, Catherine E. Davey, Kathryn S. Stok

**Affiliations:** Department of Biomedical Engineering, The University of Melbourne, Parkville, VIC 3010 Australia

**Keywords:** Rigid registration, Bone, Microarchitecture, Mechanics, Similarity measurement

## Abstract

**Purpose of Review:**

Rigid image registration is an important image processing tool for the assessment of musculoskeletal chronic disease. In this paper, we critically review applications of rigid image registration in terms of similarity measurement methods over the past three years (2019–2022) in the context of monitoring longitudinal changes to bone microstructure and mechanical properties using computed tomography. This review identifies critical assumptions and trade-offs underlying different similarity measurement methods used in image registration and demonstrates the effect of using different similarity measures on registration outcomes.

**Recent Findings:**

Image registration has been used in recent studies for: correcting positional shifts between longitudinal scans to quantify changes to bone microstructural and mechanical properties over time, developing registration-based workflows for longitudinal assessment of bone properties in pre-clinical and clinical studies, and developing and validating registration techniques for longitudinal studies.

**Summary:**

In evaluating the recent literature, it was found that the assumptions at the root of different similarity measures used in rigid image registration are not always confirmed and reported. Each similarity measurement has its advantages and disadvantages, as well as underlying assumptions. Breaking these assumptions can lead to poor and inaccurate registration results. Thus, care must be taken with regards to the choice of similarity measurement and interpretation of results. We propose that understanding and verifying the assumptions of similarity measurements will enable more accurate and efficient quantitative assessments of structural changes over time.

**Supplementary Information:**

The online version contains supplementary material available at 10.1007/s11914-023-00795-6.

## Introduction

Musculoskeletal diseases are defined by the World Health Organization (WHO) as being of long duration, taking decades to progress and whose high prevalence presents an increasing burden on both the national and family economies [1, 2]. Quantitatively assessing and tracking bone microstructure and bone mechanical properties can support diagnosis [3], monitoring [4], and establishment of treatment options [5]. To this end, 3D imaging modalities such as computed tomography (CT) and its related imaging techniques such as micro-computed tomography (microCT) [6], high-resolution peripheral quantitative computed tomography (HR-pQCT) [7], and cone-beam computed tomography (CBCT) [8] have been deployed. Conceptually, longitudinal analysis of bone microstructure and mechanical properties can be achieved by simply superimposing common volumes of interest (VOI) from images taken at different time points [9]. Nevertheless, in practice, it is difficult to control positioning shifts between longitudinal scans, even if scans were conducted by experienced and skilled imaging technologists. Mismatches related to repositioning between scans over time can result in different VOIs and inaccurate results, if not corrected.

Rigid, intensity-based, image registration is a principal tool to determine the optimal correspondence between two or more images of the same scene taken at different time points, from different viewpoints, or by different imaging modalities to locate matching VOIs over longitudinal datasets [10]. It can identify and reduce repositioning shifts introduced between scans and thus improve the precision and reproducibility of detected longitudinal changes. This is achieved by optimising the similarity between two images which can be measured in a number of ways [11•]. Popular similarity measurements that are used for image registration in the current literature are: sum of squared differences (SSD), correlation coefficient (CC), and mutual information (MI). SSD is computationally simple and assumes that the sum of mean and variance of the images is the same during the optimisation process [12•]. CC can eliminate global variations, that are introduced during scanning, by standardising the images to zero mean and unit variance [13•]. The assumption associated with CC depends on the chosen implementation of correlation [11•]. MI and its derived measures are suitable for cross-modality registration since they only assume that tissues in one image can be mapped to the same tissue in the other using approaches based on probability and information theory [14•, 15•].

In the last three years (2019–2022), the application of CT-based rigid image registration in longitudinal musculoskeletal studies has been primarily focussed on three overarching objectives: (i) applying registration to correct for repositioning shifts to reveal changes to bone microstructure [16–19] and mechanical properties over time [19–22]; (ii) development of registration-based workflows for longitudinal assessment of bone properties in pre-clinical [23, 24] and clinical studies [25•, 26, 27]; and (iii) development and validation of registration techniques for longitudinal research [28•, 29–31].

Given the growing interest in using CT-based methods for longitudinal assessment of bone quality in chronic disease, it is necessary to compare the available similarity measurements, each with inherent assumptions, so that informed decisions can be made. The primary objective of this paper is to review the application of image registration and its similarity measurement methods used in the CT field from 2019 to 2022 and identify critical assumptions and trade-offs underlying each similarity measurement method. This review demonstrates how assumptions for each similarity measures can be checked and how these assumptions may affect registration results if not considered. The focus of this review is on techniques used in recent CT literature, where frequently used algorithms are also detailed and described, where appropriate. To avoid ambiguity, in this study, image registration refers to rigid image registration only.

## Application of Image Registration in Longitudinal CT Studies

**Fig. 1:**
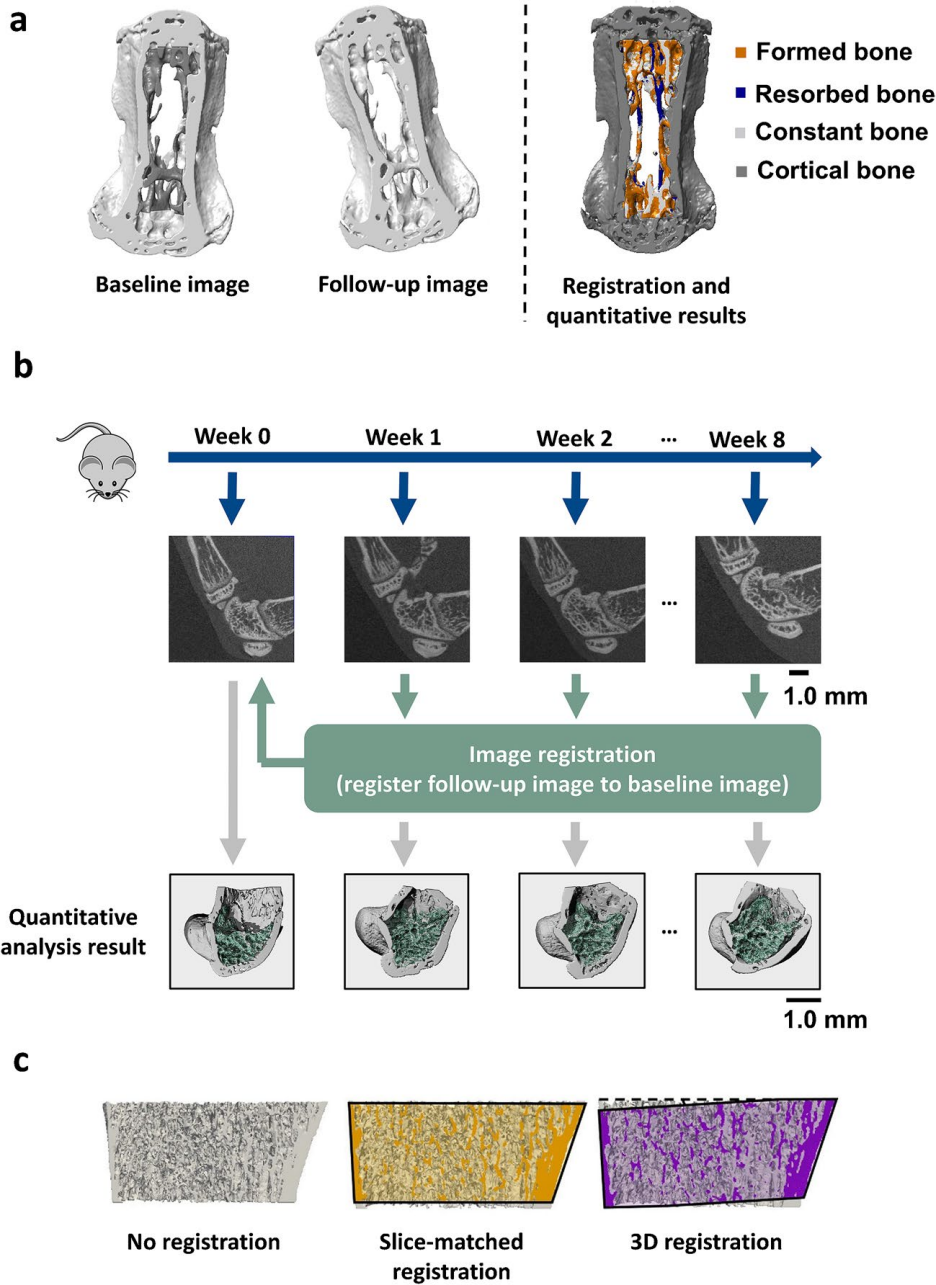
**A** Images describing the application of image registration to correct repositioning shifts for quantitative assessments of bone properties. In a longitudinal mechanical loading study, the baseline image (left) and follow-up image (central) were registered, with the registration result (right) showing formed bone (orange), resorbed bone (blue), constant bone (light grey), and cortical bone (dark grey) (*Images modified from Schulte* et al*. *[54]). (**b**) Schematic illustrating a registration-based workflow developed for longitudinal quantification of murine bone microstructural changes *(Authors' images)*. (**c**) Images showing the comparison of three segmented bone volumes used for finite element analysis of the human distal radius using no registration (left), slice-match registration (central), and 3D registration (right). [22] (*Reproduced from Plett* et al. *with permission from Springer*)

The growth in computational speed and accuracy has led many authors to incorporate image registration in their studies. Sixteen publications between 2019 to 2022 utilising image registration in longitudinal studies are reviewed and listed in Table 1 according to the study objective, along with similarity measurements. Literature is categorised according to three identified applications of image registration in the field (Fig. 1): (i) correcting repositioning shifts for quantitative assessments of bone properties; (ii) development of registration-based workflows; and (iii) development and validation of registration techniques.

**Table 1:** Applications using image registration in CT field for longitudinal study to track bone changes

Author	Study objective	Imaging modality	Scan site	Type of disease	Study length	Scan interval (total number of scan/sample*)	Isotropic voxel size	Similarity measure	Quantitative bone parameters	Registration evaluation
Zhang et al. [16]	Correct repositioning shifts	microCT	Mouse tibia	-	4 weeks	1,3 weeks (3)	10.4 µm	SSD	Cortical surface geometry	-
Brunet et al. [17]	Correct repositioning shifts	HR-pQCT	Human metacarpophalangeal joint	Early inflammatory arthritis	12 months	12 months (2)	82 µm	CC	Bone mineral density, joint space microstructure, bone erosion presence and volume	Precision error (CV%RMS, and RMSSD)
van Rietbergenen et al. [18]	Correct repositioning shifts	HR-pQCT	Human distal radius and distal tibia	-	7 years	3.5 or 7 years (2 or 3)	82 µm	CC	Periosteal expansion and retraction, endosteal expansion and retraction, cortical bone microstructure	Overlap percentage
Heilmeier et al. [19]	Correct repositioning shifts	HR-pQCT	Human ultradistal tibia and radius	Type 2 diabetes	5 years	5 years (2)	82 µm	NMI	Bone mineral density, trabecular bone microstructure, cortical bone microstructure, bone strength	-
Du et al. [20]	Correct repositioning shifts	HR-pQCT	Human distal tibia	Effect of high-impact exercise	6 months	6 months (2)	82 µm	NMI	Trabecular bone microstructure, bone stiffness	Precision error (CV%RMS)
Tourolle né Betts et al. [21]	Correct repositioning shifts	microCT	Mouse femur	Femur osteotomy	6 weeks	1 week (7)	10.5 µm	SSD	Bone volume fraction, bone resorption rate, bone formation rate, bone strain	-
Plett et al. [22]	Correct repositioning shifts	HR-pQCT	Human radius	-	3 years	6, 12 months (5)	61 µm	CC	Bone stiffness, failure load, von Mises stress	Precision error (CV%RMS), standard deviation of the absolute rate of change
Wehrle et al. [23]	Develop registration based workflow	MicroCT	Mouse femur	Femur osteotomy	6 weeks	1 week (7)	10.5 µm	SSD	Bone volume fraction, bone resorption rate, bone formation rate	-
Ning et al. [24]	Develop registration-based workflow	microCT	Rat tibia	-	5 days	5 days (2)	18 µm	MI	Bone volume fraction, trabecular bone microstructure, bone growth distance, and rate	Visual inspection, percentage of non-matching voxels
Brunet et al. [25•]	Develop registration-based workflow	HR-pQCT	Human metacarpophalangeal joint	Rheumatoid arthritis	6 months	6 months (2)	82 µm	CC	Bone volume formation fraction, bone volume resorption fraction	-
Verhelst et al. [26]	Develop registration-based workflow	CBCT	Human mandibular condyle	-	6 months	1 week, 6 months (3)	0.3 mm^3^	MI	-	ICC for inter- and intra-operator agreement
Atkins et al. [27]	Develop registration-based workflow	HR-pQCT	Human radius	-	12 months	2, 7 weeks, 3, 6 months (6)	60.7 µm	SSD	Bone mineral density, trabecular bone microstructure and cortical microstructure, formation and resorption volume fractions	-
Chiba et al. [28•]	Develop and validate registration techniques	HR-pQCT	Human distal radius, and tibia	-	4 weeks	1,3 week2 (3)	60.7 µm	SSD	Bone mineral density, trabecular bone microstructure, and cortical bone microstructure	Precision error ( CV%RMS)
Hosseinitabatabaei et al. [29]	Develop and validate registration techniques	HR-pQCT	Human radius and tibia	Osteogenesis imperfecta	1 day	1 day (2)	82 µm	CC	Bone mineral density, trabecular bone microstructure and cortical bone microstructure, bone stiffness, failure load	Precision error (CV%RMS)
Kemp et al. [30]	Develop and validate registration techniques	HR-pQCT	Human radius, and tibia	-	3 years	6, 12 months (5)	61 µm	CC	Bone mineral density, trabecular bone microstructure and cortical bone microstructure	Precision error (CV%RMS), standard deviation of the absolute rate of change
Koide et al. [31]	Develop and validate registration techniques	CT	Human spine	-	Post-operative	Post-operative (2)	1.074 mm	NMI	-	NMI improvement and Pearson's correlation

*CT* computed tomography, *microCT* micro computed tomography, *HR-pQCT* high-resolution peripheral quantitative, *CT* CBCT cone-beam CT, *SSD* sum of squared differences, *CC* correlation coefficient, *MI* mutual information, *NMI* normalised mutual information, *CV%RMS* root mean squared coefficient of variation, *RMSSD* root mean square standard deviation

^*^ Baseline scan inclusive

### Correcting Repositioning Shifts for Quantitative Assessments of Bone Properties

Bone microstructural properties (e.g., porosity, thickness [32]) and mechanical properties (e.g., stiffness, failure load [33]) play an important role in the progression of chronic musculoskeletal diseases such as osteoarthritis [34] and osteoporosis [35]. Correcting for repositioning shifts using image registration allows for accurate quantitative tracking of these changes which can provide insights into disease progression and establish markers for treatment options [20].

Many studies have used registration to uncover changes to bone microstructure over time. Zhang et al*.* [16] explored cortical bone changes in mouse tibia at multiple temporal and spatial frequencies by performing a wavelet-based analysis method on registered microCT scans using SSD. Brunet et al*.* [17] used registration based on CC to track the development of cortical erosion in early inflammatory arthritis using HR-pQCT scans of human metacarpophalangeal joint. Van Rietbergen et al*.* [18] applied CC to register follow-up grayscale HR-pQCT images of human radius and tibia from an elderly cohort and quantified changes to the endosteal contours. Heilmeier et al*.* [19] performed registration based on normalised MI to characterise 5-year changes in distal tibial and radial microstructures in postmenopausal women using HR-pQCT. Du et al*.* [20] also successfully used image registration using MI to examine localised trabecular microstructural changes in the distal tibia of postmenopausal women after a regimen of six-month regular hopping exercise.

Other studies have focused on using registration to correct for repositioning shifts prior to longitudinal finite element analysis to reveal changes in bone mechanical properties. Tourolle né Betts et al*.* [21] applied registration using SSD to a microCT dataset of mouse femur prior to evaluating changes to mechanical property over the bone fracture healing process. Plett et al*.* [22] performed image registration using CC to longitudinally assess bone strength from HR-pQCT scans of human radius. Their findings indicated an association between local mechanical strains and the formation of mineralised tissue at defect sites.

These studies have shown that registration is a useful tool for correcting longitudinal repositioning shifts and allowing for sensitive quantification of bone changes at multiple sites. They were focussed on revealing biological changes overtime; however, there are further works focussing on developing registration-based workflows with a goal to quantify these biomechanical changes.

### Development of Registration-Based Workflow for Longitudinal Research

To obtain sensitive and accurate longitudinal measurements of bone microstructural and mechanical properties, studies have proposed and evaluated acquisition and processing workflows incorporating image registration.

Investigating the effect of the microCT scanning protocol and radiation dose on bone remodelling over time, Wehrle et al*.* [23] registered successive scans of the mouse femur by applying SSD to grayscale images, and found that the protocol did not significantly interfere with bone formation and resorption. Ning et al*.* [24] provided a non-invasive microCT-based method to quantify bone growth in rat tibia, where image registration using MI is adopted to register cortical and trabecular bone, respectively. To reduce the impact of artifacts caused by stack shift from patient movement during image acquisition with HR-pQCT, Brunet et al*.* [25•] developed a multi-stack registration tool based on CC to reduce the number of discarded scans in their longitudinal studies of rheumatoid arthritis in metacarpophalangeal joint. Verhelst et al*.* [26] proposed an image processing workflow involving registration using MI for analysing human mandibular condyle using CBCT scans. Atkins et al*.* [27] developed and applied an analysis tool based on image registration for HR-pQCT scans to investigate local bone formation and resorption during fracture healing in human radius. 

Studies with this objective were mainly focussed on applying existing registration routines into wider workflows for longitudinal assessment of bone [23, 24, 25•, 26, 27]. There are also a number of other works that are actively contributing to the improvement and validation of existing registration techniques [28•, 29–31].

### Development and Validation of Registration Techniques

In recent years, many image registration techniques in use are 3D registration methods where a combination of rotation and translation operations are performed [28•, 29, 30]. However, a computationally simpler approach to image registration can also be performed in a slice-matched manner, where only translation is included [36]. Chiba et al*.* [28•] compared the variation in quantitative bone measurements between 3D registration using SSD and slice-matched registration with binarised HR-pQCT scans of healthy human radius and tibia. Their results showed that 3D registration yielded lower variation for measurements of bone mineral density and microstructural parameters between follow-up scans. Hosseinitabatabaei et al*.* [29] and Kemp et al*.* [30] reached a similar conclusion after investigating the effect of 3D registration using CC and slice-matched registration on the precision of bone microstructural and mineral density measurements in human radius and tibia using greyscale HR-pQCT datasets.

In a separate development, Koide et al*.* [31] implemented and compared an automated registration using normalised MI with manual registration workflow in clinical planning CT and post-operative contrast-enhanced CT of the human spine. The study found comparable outcomes between both approaches, suggesting that automated registration using MI for surgical planning could be an efficient alternative to manual registration. Notably, semi-automatic techniques—also available and prevalent—often also suffer from efficiency and reproducibility issues as observed by Koide et al*.* [31] with manual image registration. Fully automatic techniques are still preferred wherever applicable.

There are many studies employing image registration for longitudinal applications, and with an increasing interest in acquisition of longitudinal CT data in bone research, there is a need to critically evaluate the different similarity measurement techniques used for image registration and their suitability for the research question. In the following sections, a mathematical introduction to image registration and the fundamental underlying assumptions associated with each commonly used similarity measurement is presented.

## Assumptions in Similarity Measurements for Registration

A CT image can be converted into a 2D matrix by stacking slices of data along one specific dimension (typically stacked along the z-dimension) [37], where each element contains an intensity value from a single voxel. In their 2D matrix forms, consider the baseline image, ${\varvec{U}}$, and the follow-up image with the same dimension, ${\varvec{V}}$. The objective of image registration is to perform transformation, $T$, such that the difference between the baseline image, ${\varvec{U}}$, and the transformed follow-up image, $T({\varvec{V}})$, is minimised. This can be achieved by optimising a measure of similarity, $c$, between the baseline image, ${\varvec{U}}$, and the transformed follow-up image, $T\left({\varvec{V}}\right)$, to obtain the optimal transformation, ${T}_{opt}$:

1 $$T_{opt}=\mathit{arg}\mathit\;\underset T{\mathit{min}}\;c\left(\boldsymbol U,T\left(\boldsymbol V\right)\right)$$

Intensity-based registration methods utilise voxel information from the images for the computation of similarity measure, $c$. As mentioned in earlier sections, there are several approaches for measuring similarity [38]. The most widely used measures, including SSD, CC, and MI, are described here.

### Sum of Squared Differences

Similarity measure based on SSD, ${c}_{SSD}$, has been widely used in [16, 21, 23, 28•] and is defined as follows:

2 $${c}_{SSD}=\begin{array}{cc}{\sum }_{i}^{N}{({u}_{i}-{T\left(v\right)}_{i})}^{2}& \in [0,\infty )\end{array}$$

3 $$={\sum }_{i}^{N}{{u}_{i}}^{2}+{\sum }_{i}^{N}{{T\left(v\right)}_{i}}^{2}-2{\sum }_{i}^{N}{u}_{i}{T\left(v\right)}_{i}$$

4 $$=N{\sigma }_{u}^{2}+N{\overline{u} }^{2}+N{\sigma }_{T\left(v\right)}^{2}+N{\overline{T\left(v\right)^{2}}}-2N\rho$$

where $u$ and $T\left(v\right)$ are the scalar forms of CT image, ${\varvec{U}}$, and transformed image, $T\left({\varvec{V}}\right)$. $N$ denotes the total number of voxels in each image and $\rho$ represents correlation, $\sum_{i}^{N}{u}_{i}{T\left(v\right)}_{i}$, between ${\varvec{U}}$ and $T\left({\varvec{V}}\right)$. Mean and variance of ${\varvec{U}}$ and $T({\varvec{V}})$ are written as $\overline{u }$, ${\sigma }_{u}^{2}$, $\overline{T\left(v\right)}$, and ${\sigma }_{T\left(v\right)}^{2}$, respectively, and can be calculated directly from each image. The optimal value of ${c}_{SSD}$ is the value that has the smallest magnitude (i.e., smallest difference between ${\varvec{U}}$ and $T\left({\varvec{V}}\right)$).

Equation (4) shows multiple terms summing up to calculate${c}_{SSD}$, among which, correlation,$\rho$, is the only element that measures the similarity between baseline and transformed follow-up image, while other terms,$\overline{u }$,${\sigma }_{u}^{2}$,$\overline{T\left(v\right)}$, and${\sigma }_{T\left(v\right)}^{2}$, are their inherent properties. For ${c}_{SSD}$ to be accurate in measuring similarity between two images, ${c}_{SSD}$ should be only proportional to correlation,$\rho$. As baseline, $\overline{u }$ and ${\sigma }_{u}^{2}$ are constant throughout the optimisation search, which is the process of searching for minimum of${c}_{SSD}$. However, it should be noted that during optimisation search, the value of$\overline{T\left(v\right)}$, and${\sigma }_{T\left(v\right)}^{2}$, can be altered. During rotational and translational search, the transformed image may have to be cropped and padded to maintain consistent image dimension. Depending on the cropping and padding approaches, this can lead to changing$\overline{T\left(v\right)}$, and${\sigma }_{T\left(v\right)}^{2}$, in addition to the already changing correlation,$\rho$. Thus, to ensure that ${c}_{SSD}$ reflects the changing $\rho$ during the optimisation process, the sum of $\overline{T\left(v\right)}$ and ${\sigma }_{T\left(v\right)}^{2}$ should be constant throughout, which is the key assumption in the SSD method.

Small deviations from this assumption should be tolerable but will introduce more registration errors. These deviations from assumption and registration errors should be discussed when interpreting the quantitative analysis results. Additionally, the presence of severe outliers, such as may result from intensity variation introduced by use of contrast agents, or extensive bone remodelling, can lead to substantially larger deviation in $\overline{T\left(v\right)}$ and ${\sigma }_{T\left(v\right)}^{2}$ [12•]. In the worst scenario, outliers can cause a significant shift in $\overline{T\left(v\right)}$ and ${\sigma }_{T\left(v\right)}^{2}$ during optimisation search, such that the calculated transformation that optimises ${c}_{SSD}$ is different from the transformation that optimises the correlation between the images. To avoid this, it is recommended to maintain constant sum of $\overline{T\left(v\right)}$ and ${\sigma }_{T\left(v\right)}^{2}$ during an optimisation search. This can be achieved by employing periodic boundary padding [39] or by considering alternative similarity measures. 

Of the five studies under review that apply SSD-based registration, Zhang et al*.* [16], Tourolle né Betts et al*.* [21], Wehrle et al*.* [23], and Atkins et al*.* [27] assumed sufficiently accurate rigid registration to not discuss registration as a potential source of error when reporting results. Chiba et al*.* [28•] mentioned inaccuracies in their trabecular bone resorption and formation results and recognised that imperfect registration could be a contributor. In this work [28•], registration error was attributed to acquisition issues such as motion artifacts, noise, and instability of the CT device. Additionally, their registration issues could possibly stem from the use of binary, rather than greyscale, images for registration, which could lead to inaccurate results due to loss of information during the binarisation process [40].

None of the five SSD studies considered in this report verified that their data satisfied the mean and variance assumption implicit in SSD (see Eq. (4) to be clear). These studies did not use contrast agent and saw no substantial bone remodelling; consequently, the assumption was likely satisfied. However, by not validating adherence to the assumption underlying SSD, their quantitative results may be affected by inaccuracies in the registration.

SSD can be advantageous as it is computationally efficient, with an $O\left(n\right)$ complexity, which indicates that the number of operations required to calculate the similarity measure grows proportionally with image size, *n*. However, the implicit assumption that the mean and variance remain constant can be easily violated. Rotating an image during optimisation search changes the voxels that fall within the image frame, impacting estimation of the mean, $\overline{T\left(v\right)}$, and variance, ${\sigma }_{T\left(v\right)}^{2}$, of the transformed follow-up image. This may affect the similarity measure calculated for different rotations, potentially causing inaccurate registration and impacting microstructural measurement results. To ensure reproducibility across trials and different registrations, it is critical to ensure that precision error of image registration falls within specified confidence intervals. Glüer et al*.* [41] provide guidance on how to calculate precision error for user specified confidence intervals specifically for bone densitometric techniques.

### Correlation Coefficient

Correlation based approaches, denoted CC, use a measure of linear association between a baseline image, ${\varvec{U}}$, and a transformed image, $T\left({\varvec{V}}\right)$, to determine similarity, ${c}_{CC}$. The assumption associated with ${c}_{CC}$ therefore depends on the chosen implementation of correlation. There are a number of correlation measures utilised for registration in the literature, the most common being Pearson’s correlation [42•],

5 $$\begin{array}{cc}c_{CC}=\frac{\sum_i^N\left(u_i-\overline u\right)\cdot\left({T\left(v\right)}_i-\overline{T\left(v\right)}\right)}{\sigma_u\sigma_{T(v)}}&\in\left[-1,1\right]\end{array}$$

In this form, correlation measures linear association between baseline image, ${\varvec{U}}$, and transformed image, $T\left({\varvec{V}}\right)$, and has $O(n)$ complexity. Correlation standardises the inputs to zero mean and unit variance, so that it is robust to changes in image intensity. Correlation makes an implicit assumption that the relationship between the two images is linear [43]. The optimal value of ${c}_{CC}$ is given by the transformation resulting in the input images having the strongest linear association.

The strength of the linear relationship can be determined by directly calculating correlation between two images using multiple methods, including but not limited to Spearman’s correlation [42•] and Kendall’s tau [44]. Several reviewed studies utilized commercial image processing platform IPL (SCANCO Medical AG, Brüttisellen, Switzerland) to perform image registration using CC in their longitudinal studies [17, 18, 22, 29, 30]. Additionally, toolboxes are also available in MATLAB (MathWorks, Natick, MA, USA) [45], along with other cost-free packages in ITK [24], and Python [46]. All image registration using CC considered in this review, as well as most available registration toolboxes, employed Pearson’s correlation [17, 18, 22, 25•, 29, 30]. 

None of the studies reviewed discussed the linearity assumptions. Brunet et al*.* [25•] acknowledged that slight errors in registration may lead to inaccuracies in the results. Even though CC is widely accessible, the exact correlation approach and their assumptions should always be checked (e.g., performing Spearman’s correlation or Kendall’s tau) before implementation. A demonstration of how these assumptions can be checked is available in the following section “Demonstration of critical assumptions on registration results from different similarity measures” and the supplementary material of this manuscript. 

### Mutual Information

MI-based registration has gained much popularity since it was performed by Viola et al*.* [14•] and Maes et al*.* [15•] as it only assumes that one tissue in the baseline image can be mapped to the same tissue in the follow-up image. Instead of performing voxel-size evaluation of similarity as is done in SSD and CC, MI use techniques from information theory to calculate the amount of information one image contains about the other [47].

For this section, the baseline and transformed follow-up images are considered as random variables, $U$ and $T(V)$. Their Shannon entropy can be written as $H\left(U\right)$ and $H\left(T(V)\right)$ [48], where:

6 $$H(U)=-\sum\nolimits_{m\in U}{logP}_{U}(m){P}_{U}(m),$$

7 $$H(T(V))=-\sum\nolimits_{n\in T(V)}{logP}_{T(V)}(n){P}_{T(V)}(n),$$

with ${P}_{U}(m)$ being the probability that a voxel in image $U$ would have an intensity $m$, and ${P}_{T(V)}(n)$ being the probability that a voxel in image $T(V)$ would have an intensity $n$. A joint histogram of the two images can be used to estimate a joint probability distribution [47]. The Shannon entropy, $H\left(U,T(V)\right)$, of the joint distribution can be, then, written as:

8 $$H(U,T(V))=-\sum\nolimits_{m\in U,n\in T(V)}{logP}_{U,T(V)}(m,n){P}_{U,T(V)}(m,n),$$

where ${P}_{U,T(V)}\left(m,n\right)$ is the joint probability that a voxel with intensity $m$ in the image $U$ correspond to intensity $n$ in the image $T(V)$.

With the terms described, similarity measurement using MI, ${c}_{MI}$, can be defined as:

9 $$\begin{array}{cc}{c}_{MI}=H\left(U\right)+H\left(T(V)\right)-H\left(U,T(V)\right)& \in \left[0,\left.\infty \right)\right.\end{array}$$

The magnitude of MI is dependent on $H\left(U\right)$, $H\left(V\right)$, and $H\left(U,T(V)\right)$, ranging from 0 to infinite, where an optimised MI value has the largest magnitude. However, the magnitude of ${c}_{MI}$ can be unintuitive and difficult to interpret due to its unbounded range. Kvalseth et al*.* [49] proposed normalised mutual information (NMI) to enable comparison by scaling ${c}_{MI}$ to a bounded range, defined as the ratio between mutual information and sum of entropy of two images:

10 $$\begin{array}{cc}{c}_{NMI}=\frac{2{c}_{MI}}{H\left(U\right)+H\left(T(V)\right)}& \in \left[\mathrm{0,1}]\right.\end{array}$$

Mutual information can also be normalised using other quantities, such as, joint entropy [50]. In this review, two studies [24, 26] used MI and three used NMI [19, 20, 31] as their similarity measure to perform registration.

It is worth noting that using MI-based similarity measure is computationally more complex than SSD and CC. With complexity of $O(nlogn)$ [51], MI can introduce efficiency and memory issues in longitudinal registration of large datasets using high resolution scans where SSD and CC, with $O(n)$ complexity, could have achieved the same result with fewer resources. In fact, no study using MI-based registration in this review incorporated a large number of datasets. Compared to the study of van Rietbergen et al*.* [18] using more than 1000 HR-pQCT scans with CC for registration, the largest dataset using MI-based registration is from Ning et al. [24], with 144 microCT scans.

Table 2 provides a summary of recommendations for various scenarios, with pros and cons for each similarity measure. For scenarios involving slight changing bone structure and no contrast agent, SSD is recommended to be adopted to perform image registration due to its computational efficiency $O\left(n\right)$. On the other hand, CC is recommended when no contrast agent and rapid changing bone microstructure is involved, and high correlation between two images is ensured. The minimal assumptions introduced by MI makes it suitable for applications where rapidly changing bone microstructures or the intensity of images are shifted by the introduction of contrast agent.

**Table 2:** Summary of recommendations for various scenarios, with pros and cons for each similarity measure

Similarity measure	Scenarios	Pros	Cons
SSD	Stable bone microstructure; no contrast agent	Linear computational complexity	The strict assumption needs to be satisfied
CC	Slight changing bone microstructure; no contrast agent	Computationally efficient; robust to global variations in image intensity	Need to check correlation between two images prior to image registration
MI	Rapid changing bone microstructure; with contrast agent	The most robust	Computationally expensive

## Demonstration of Critical Assumptions on Registration Results from Different Similarity Measures

**Fig. 2:**
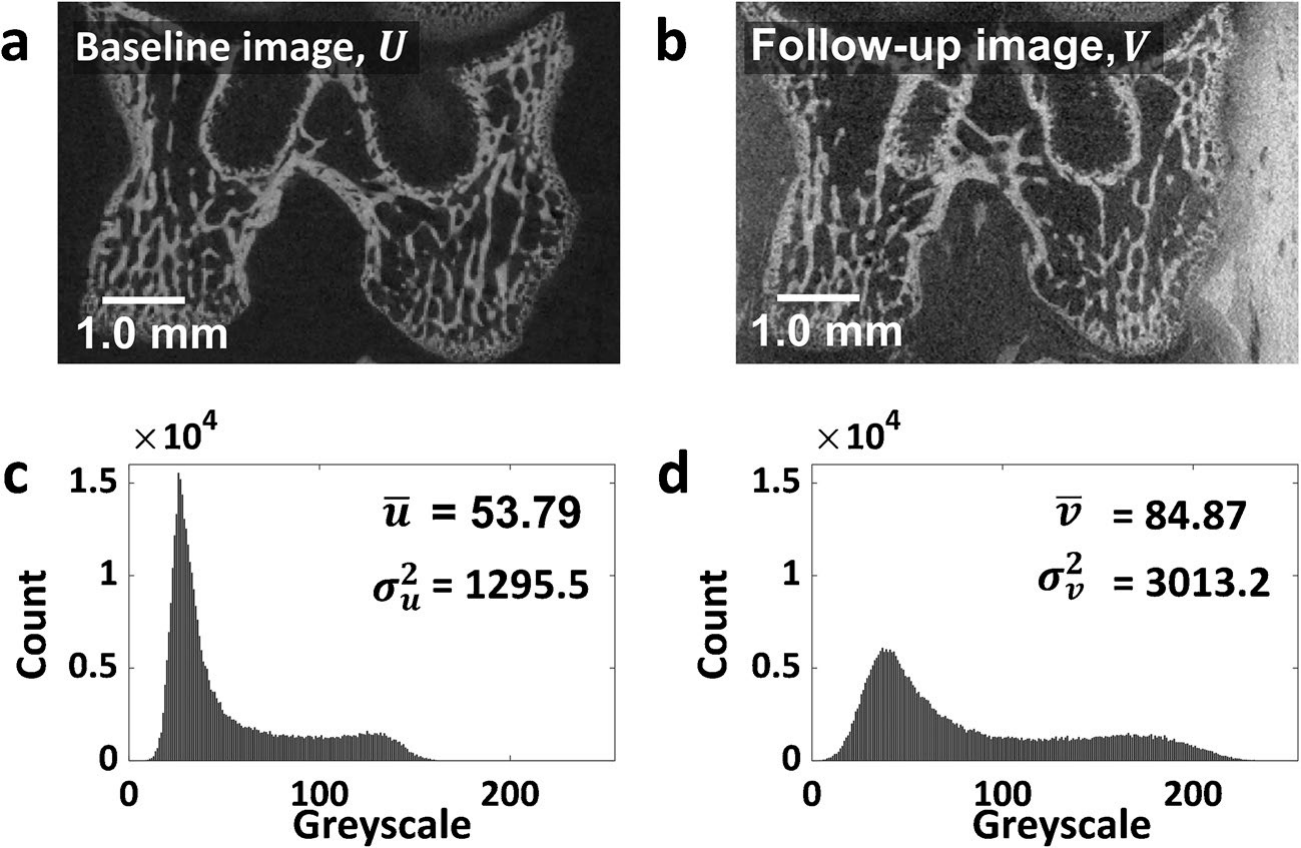
Representative 2D slices of (**a**) baseline and (**b**) follow-up microCT images of the rat femur. The follow-up image was acquired following the injection of contrast solution into the joint cavity. Histograms and summary statistics of the baseline image (**c**) and the follow-up image (**d**), including the mean and variance are also presented

To demonstrate how assumptions for each similarity measures can be checked and how they affect registration results, this section presents an example application of microCT images of the rat femur from a previous study of osteoarthritis [52]. For computational efficiency and simplicity in the presentation of results, a down-sampled 2D slice is used to present the 3D image. All operations were performed in MATLAB (R2022a, MathWorks, Natick, MA, USA). Figure 2 shows the baseline and follow-up input images, their histograms, and summary statistics. The baseline image (Fig. 2a) was acquired at the beginning of the study and the follow up image (Fig. 2b) was acquired after injecting a contrast solution into the joint cavity. The histogram and summary statistics of the baseline image (Fig. 2c) and the follow-up image (Fig. 2d) are presented. The introduction of a contrast solution has enhanced brightness and caused a substantial increase in the mean and variance of the follow-up image, resulting in a low Spearman’s correlation (0.14) between the two.

**Fig. 3:**
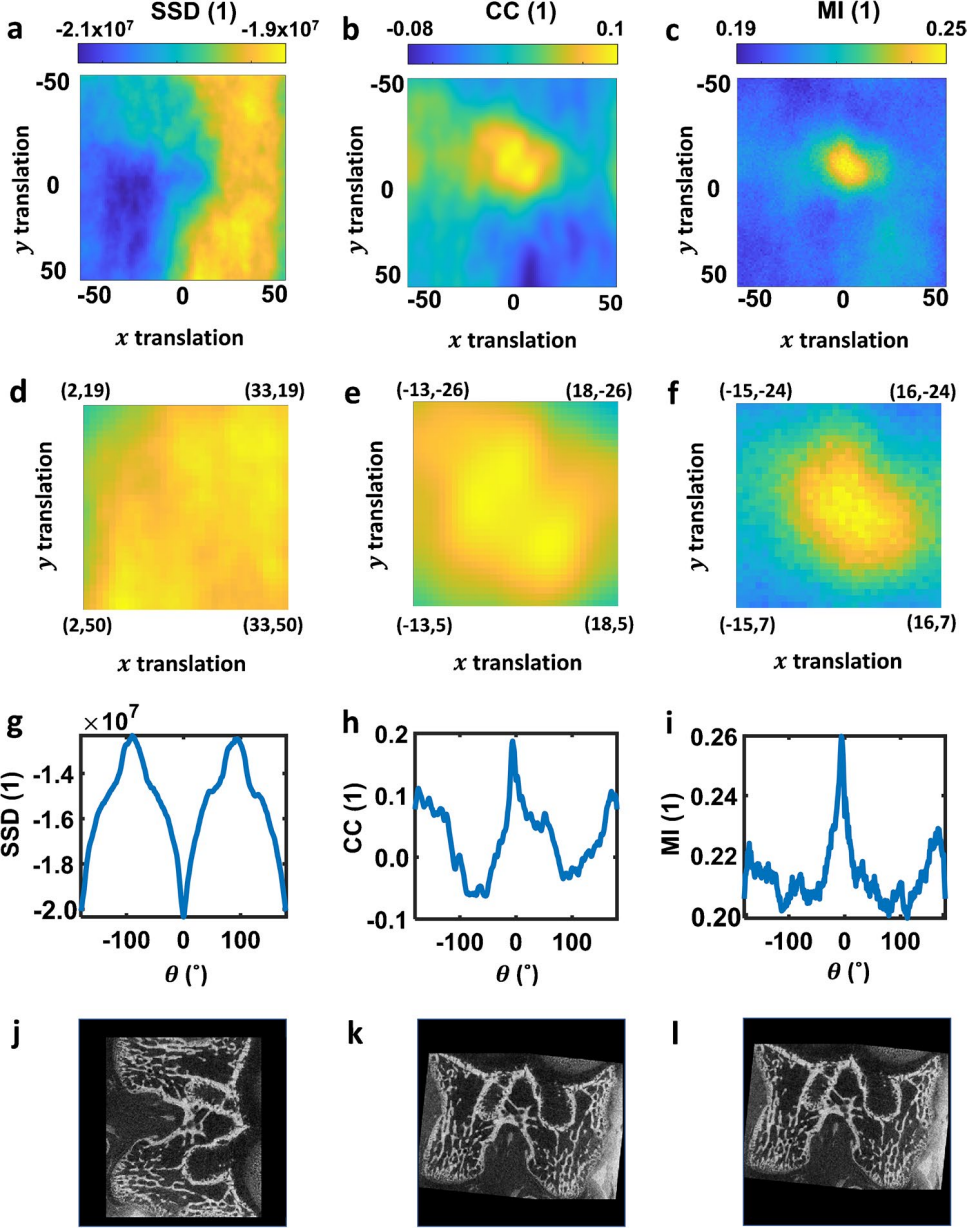
Computed similarity score and registration outcome using different similarity measurements methods. Top row: similarity scores calculated using (**a**) SSD, (**b**) CC and (**c**) MI, when translating the follow-up image from -50 to + 50 pixels along the x- and y-axis, respectively. Second row: detailed view focussing on plotting area with high similarity scores calculated using (**d**) SSD, (**e**) CC and (f) MI. Third row: similarity scores calculated using (**d**) SSD, (**e**) CC, and (**f**) MI, when rotating the follow-up image from -180˚ to 180˚. Bottom row: registration result obtained by applying the optimal translation and rotation results using (**g**) SSD, (**h**) CC, and (**i**) MI methods

A brute-force optimal transformation search was performed by translating the follow-up image from -50 to + 50 pixels along the x- and y-axis at 1 pixel increment and rotating the follow-up image from -180˚ to + 180˚ clockwise with a step size of 1˚, with translation and rotation performed independently. For each transformation, similarity measures (SSD, CC, MI) were evaluated as described and are shown in Fig. 3. The highest value for each similarity measure was selected as ‘optimised’ and the subsequent registration results presented. To achieve this, SSD scores were inverted for consistent interpretation. Since the baseline and follow-up images are already in a very similar starting position, minimal translation and rotation are expected in the registration result.

Similarity scores for translation show that SSD has detected multiple optimised points around the extremities of the translation (x = 34, y = -34, and x = 30, y = 25, Fig. 3a). Though CC (Fig. 3b) has correctly estimated that minimal translation is needed, multiple optimised points can be seen around the translation centre (0,0). While MI showed clear optimised points even with the intensity distortion caused by contrast solution (Fig. 3c). These results can be better observed in the detailed view of similarity scores calculated using SSD (Fig. 3d), CC (Fig. 3e), and MI (Fig. 3f).

Similarity scores for rotation also show that SSD has falsely detected multiple peaks at ± 100˚ rotation (Fig. 3g), while CC (Fig. 3h) and MI (Fig. 3i) have correctly approximated minimal rotation. These similarity measures are reflected in their respective registration results where registration using SSD failed (Fig. 3j) while registration using CC (Fig. 3k) and MI (Fig. 3l) registrations show good similarity to the baseline image.

These results are expected as it can be seen from the registration result for SSD (Fig. 3j), the bright areas on both sides of follow-up image (Fig. 2b) were rotated to optimise ${c}_{SSD}$ and cropped to maintain consistent image dimension, which lead to incorrect registration result. Though the low Spearman’s correlation between baseline and follow-up images translates to an approximate successful CC-based registration, the multiple peaks around the optimal extremity could cause the algorithm to be trapped in local extrema, and could lead to an inaccurate registration result. MI, having the least strict assumption, shows the sharpest optimised peak in its similarity scores (Fig. 3c, f, i) which is reflected in its registration result (Fig. 3l).

More examples and use cases can be found in the supplementary documentation. To validate the algorithm, two identical image examples are used to perform image registration. To further explore how breaking SSD assumptions can affect the registration result, a longitudinal registration example using microCT scans from a mouse tibia is also available.

## Perspectives and Conclusion

Recent literature, from 2019 to 2022, that applied image registration for the longitudinal assessment of bone microstructural and mechanical properties has been critically reviewed, where the goal and type of registration in those studies were analysed. An introduction to image registration methods reporting various similarity measures and their underlying assumptions were examined, and the effect of these assumptions on longitudinal microCT data was demonstrated.

This review found that despite the increasing popularity of image registration in longitudinal studies using CT, choices regarding similarity measures has not been regularly discussed. As shown in this review, each similarity measure has its own assumptions which, if broken, would lead to poor and inaccurate registration results. SSD is computationally simple to perform and implement but comes with a strict assumption which can be easily broken in longitudinal studies; namely the constant sum of mean and variance of the transformed, follow-up image during optimising process. CC based on Pearson’s correlation is also computationally efficient, but care must be taken to ensure high linear correlation between the images. MI is arguably the most robust of the major measures of similarity; however, it is computationally expensive. High-resolution 3D data, such as those from a mouse study of osteoarthritis [53], have x, y, and z dimensions of 3198 × 3198 × 335 voxels (3.4 × 10^9^ voxels) for a single scan. Using MI, with its $O(nlogn)$ complexity, could lead to a registration time of several days longer than either CC or SSD. To improve time-efficiency of the registration process, it is recommended to implement image registration with a with a multi-resolution or pyramid strategy [12•].

Additionally, image registration, ubiquitous in its application, is not a perfect tool. Whether performed manually or automatically, registration errors can still be introduced. Registration errors should be accounted for during study planning and appropriately discussed when reporting results.

In summary, advances in image registration have stimulated many studies to use it as a critical tool for time-lapse CT imaging to monitor changes to bone microstructure and mechanical property. The understanding and verification of the assumptions and trade-offs behind different similarity measurements will enable a more accurate and efficient quantitative measurements while appreciating the nuances of each image registration approach.

## Supplementary Information

Below is the link to the electronic supplementary material.

Supplementary file1 (DOCX 3470 KB)

## Data Availability

The authors declare that original data supporting the perspectives of this review are available within the paper and its supplementary information files.
