# Supplementary material for: Image Registration in Longitudinal Bone Assessment Using Computed Tomography
Source: Curr Osteoporos Rep. 2023 Jun 2;21(4):372–85. doi: 10.1007/s11914-023-00795-6 (PMC10393902; doi:10.1007/s11914-023-00795-6)

**Image registration in longitudinal bone assessment using computed tomography**

Han Liu^a^, Pholpat Durongbhan^a^, Catherine E. Davey^a^, Kathryn S. Stok^a*^

^a^ Department of Biomedical Engineering, The University of Melbourne, Parkville, VIC Australia

**Supplementary information**

**Supplementary figures**

**Demonstration of registration using longitudinal dataset**

Supplementary Figures S1: Representative 2D slices of (a) baseline and (b) follow-up microCT images of the mouse femur. The baseline image was acquired at the beginning of the study. The follow-up image was acquired eight weeks later. Histograms and summary statistics of the baseline image (c) and the follow-up image (d), including the mean and variance are also presented. The Spearman’s correlation between baseline and follow-up image is 0.52.


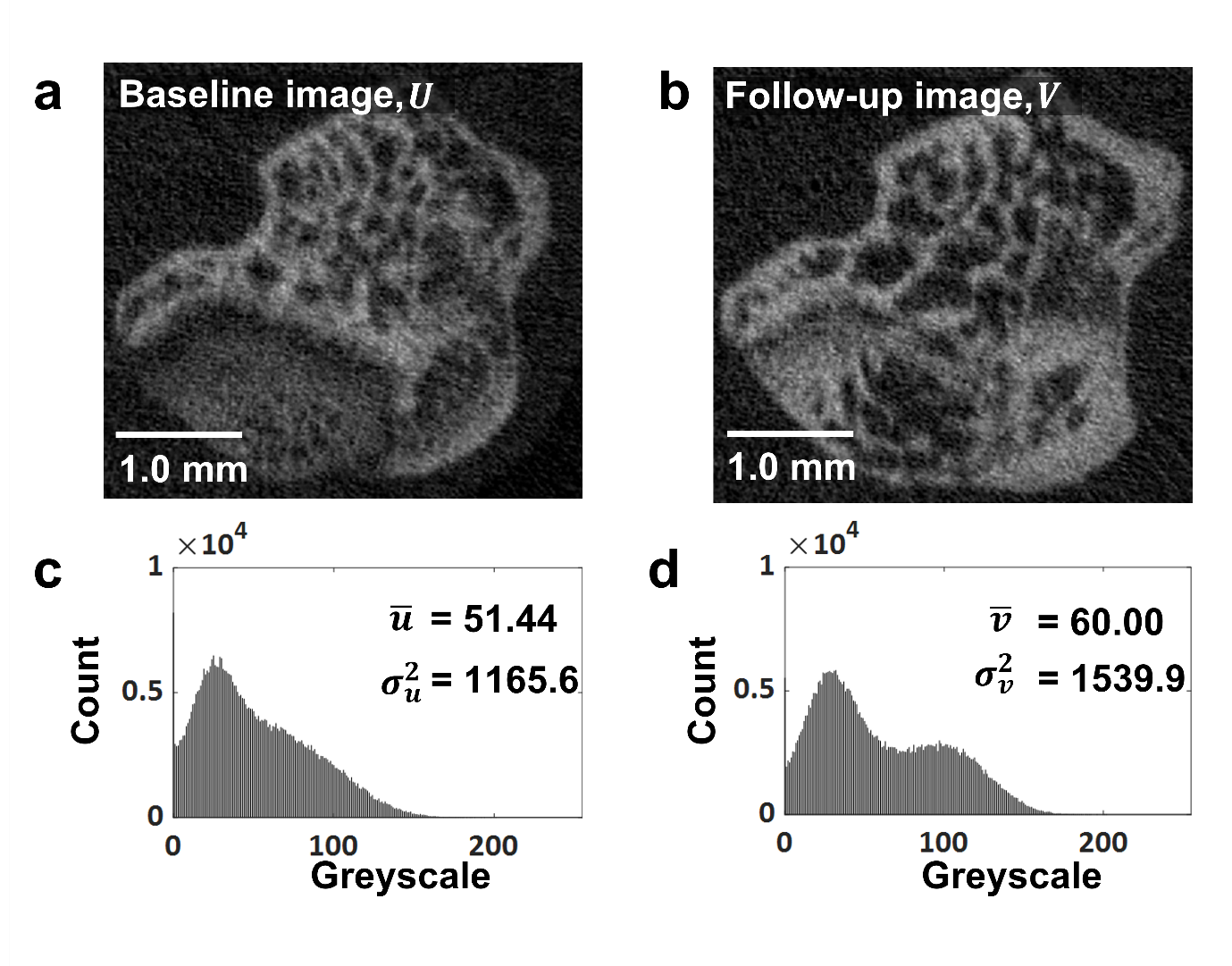


Supplementary Figures S2: Computed similarity score and registration outcome using different similarity measurements methods. Top row: similarity scores calculated using (a) SSD, (b) CC and (c) MI, when translating the follow-up image from -50 to +50 pixels along the x- and y-axis, respectively. In the middle, similarity scores calculated using SSD (d), CC (e) and MI (f), when rotating the follow-up image from -180˚ to 180˚, respectively. At the bottom, registration results obtained by applying the optimal translation and rotation results using SSD (g), CC (h), and MI (i) methods.


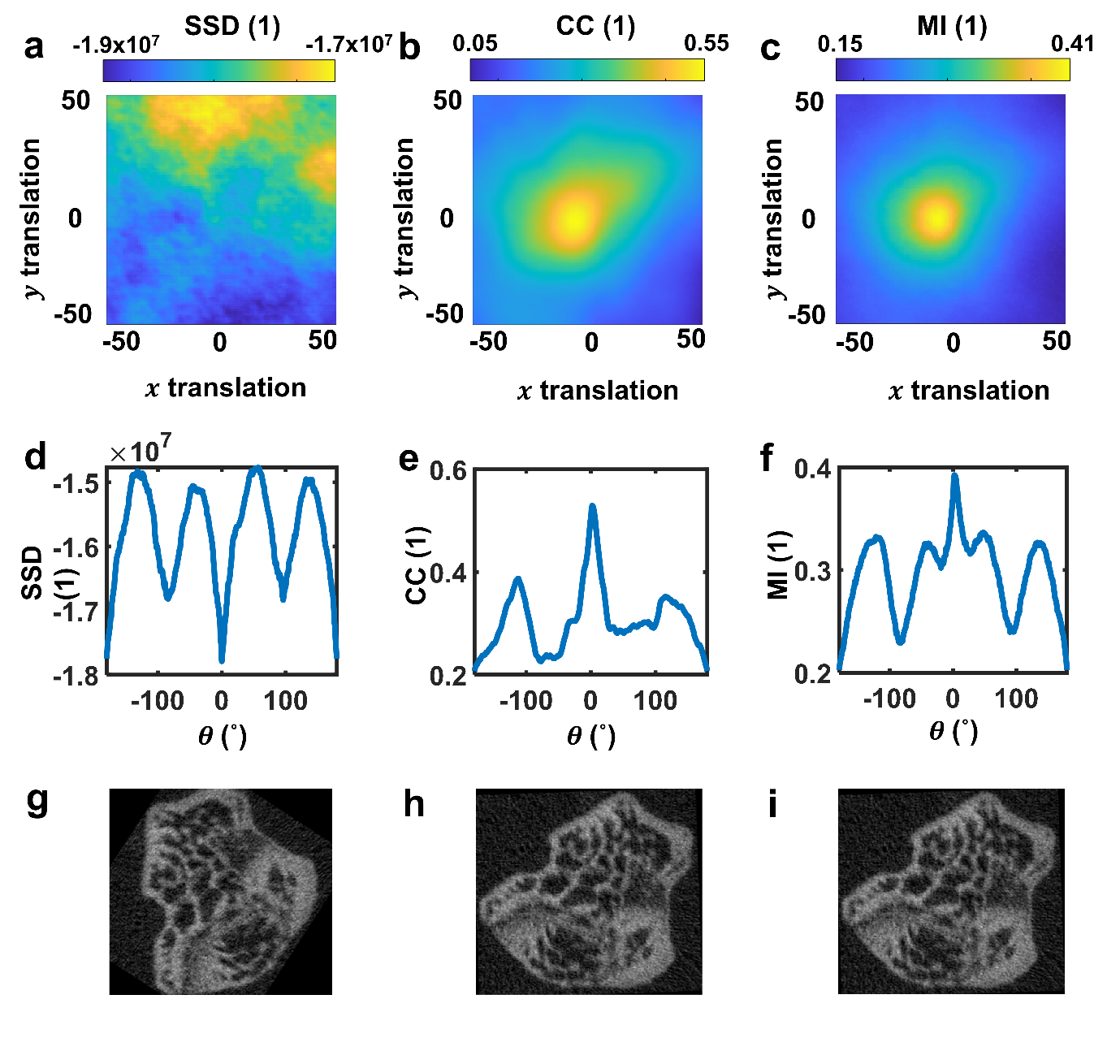


**Validation of the established algorithm using identical images**

Supplementary Figures S3: Representative 2D slices of (a) baseline and (b) follow-up microCT images of the mouse femur. The baseline image was also taken as the follow-up image to validate the established algorithm. Histograms and summary statistics of the baseline image (c) and the follow-up image (d), including the mean and variance are also presented. The Spearman’s correlation between baseline and follow-up image is 1.


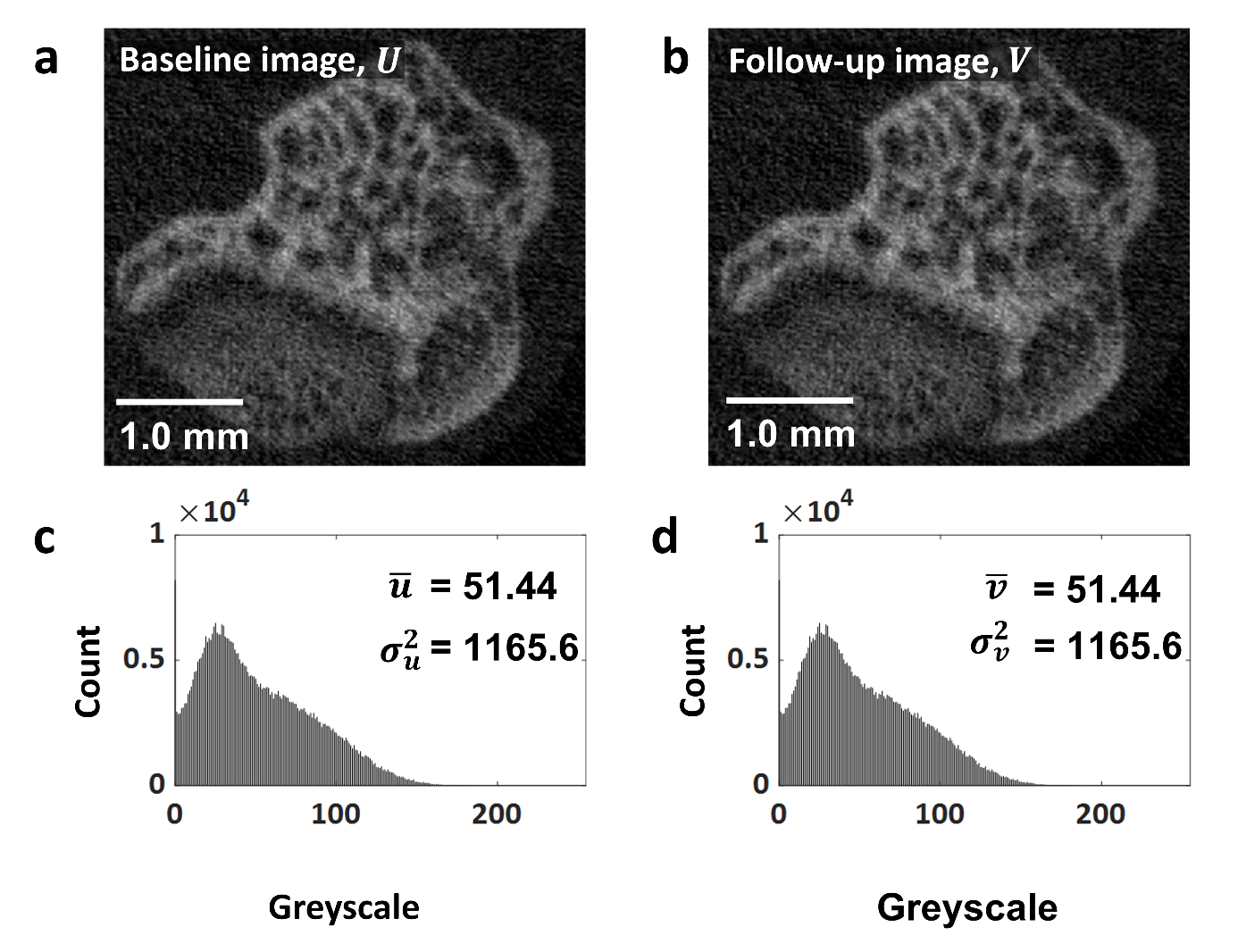


Supplementary Figures S4: Computed similarity score and registration outcome using different similarity measurements methods. Top row: similarity scores calculated using (a) SSD, (b) CC and (c) MI, when translating the follow-up image from -50 to +50 pixels along the x- and y-axis, respectively. In the middle, similarity scores calculated using SSD (d), CC (e) and MI (f), when rotating the follow-up image from -180˚ to 180˚, respectively. At the bottom, registration results obtained by applying the optimal translation and rotation results using SSD (g), CC (h), and MI (i) methods.
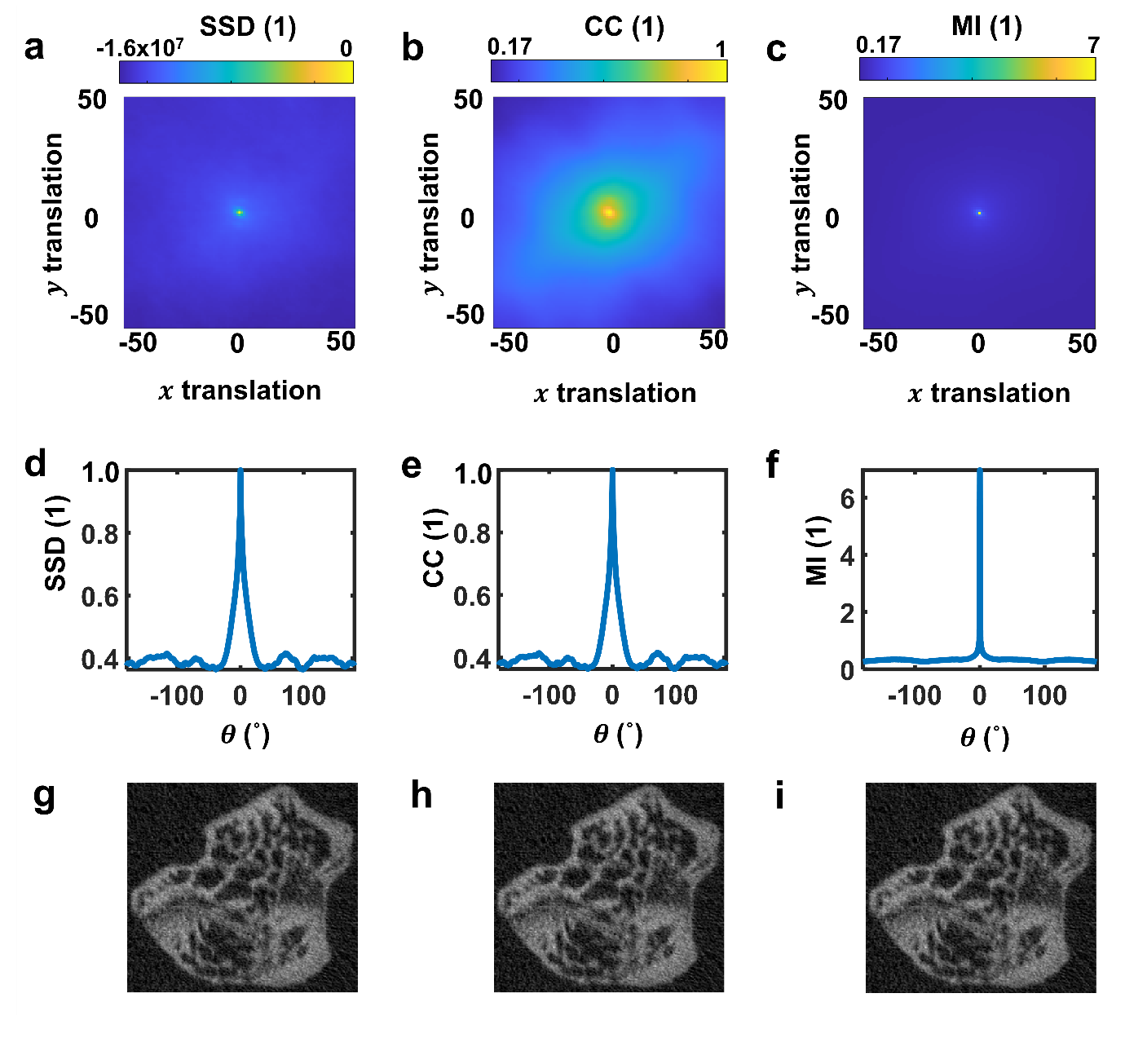

Supplement: Supplementary file 1 — Supplementary file1 (DOCX 3470 KB) [file 11914_2023_795_MOESM1_ESM.docx]
